# Hotspots of un-iodized salt availability among Ethiopian households, evidence from the national survey data

**DOI:** 10.1186/s41043-023-00359-5

**Published:** 2023-03-17

**Authors:** Atalay Goshu Muluneh, Mehari W. Mariam Merid, Getahun Molla Kassa

**Affiliations:** Department of Epidemiology and Biostatistics, Institute of Public Health, College of Medicine and Health Sciences, University of Gondar, P.O. Box 196, Gondar, Ethiopia

**Keywords:** Un-iodized salt, Hotspots, Ethiopia

## Abstract

**Background:**

Universal salt iodization was started before decades but there are communities using the un-iodized salt till now. More than one-tenth of the Ethiopian community uses un-iodized salt.

**Objective:**

This study aimed to identify the hotspots and associate factors of un-iodized salt availability in Ethiopia based on Ethiopian national household survey data.

**Methods:**

We conducted an in-depth analysis of the Ethiopian Demographic and Health Survey 2016 data. A total of 15,567 households were included in the final analysis. We cleaned and weighed the data using Stata version 16 software and descriptive outputs were reported in graphs and tables. We computed the weighted prevalence of un-iodized salt and prepared it for spatial analysis. Global-level spatial autocorrelation, hotspot analysis using the Getis-Ord Gi* statistics, and spatial interpolation using empirical Bayesian interpolation were executed using ArcGIS 10.3 to predict the magnitude of un-iodized salt at the national level. The binary logistics regression model was used to identify the contributing factors of un-iodized salt utilization. Model goodness of fit was tested with Hosmer and Lemeshow goodness-of-fit test (*P* = 0.96). Finally, the adjusted odds ratio (AOR) with 95% CI was reported to identify significant factors.

**Results:**

The magnitude of un-iodized salt availability was 14.19% (95% CI: 13.65, 14.75) among Ethiopian households. Un-iodized salt hotspots were found in Afar, Somalia, and Benishangul Gumuz regions. Compared to poorest wealth index: poorer (AOR = 0.55, 95% CI: 0.48, 0.64), middle (AOR = 0.51, 95% CI: 0.44, 0.60), richer (AOR = 0.55, 95% CI: 0.47, 0.64), and richest (AOR = 0.61, 95% CI: 0.50, 0.75); compared to uneducated household head: heads with secondary (AOR = 0.72, 95% CI: 0.60, 0.67) and above secondary (AOR = 0.54, 95% CI: 0.43, 0.67) education reduced the odds of un-iodized salt viability, while households living in highland (AOR = 1.16, 95% CI: 1.05, 1.29) had increased the odds of un-iodized salt availability.

**Conclusion:**

More than a tenth of the households in Ethiopia uses un-iodized salt. Hotspots of un-iodized salt availability were found in Somali and Afar regions of Ethiopia. Better wealth index and education of the household heads reduces the odds of un-iodized salt availability while living in a high altitude above 2200 m increases the odds of un-iodized salt availability in Ethiopia.

## Background

Iodine is one of the most deficient micronutrients and more than a quarter of the world population does not access adequate iodized salt [1]. Iodine deficiency is the most important cause of mental impairment that could be prevented by simple iodization and other public health interventions [2]. Ethiopia is one of the ten key salt-producing country in Sub-Saharan Africa [3]. Many African countries do not achieve at least 90% universal salt iodization and Ethiopia is the 3rd country with the highest rate of non-iodized salt availability in the households (HH) among sub-Saharan countries [4]. Different countries have a varied rate of iodized salt availability; 73.15% Bangladesh [5] and 69.8% in Saudi Arabia [6].The coverage of household iodized salt range from 6.2% in Niger to 97% Uganda among African regions [7] which is lower as compared to the global recommendation of > 90% of households must use iodized salt [8]. In Ethiopia, more than 10% of the households do not use iodized salt [4] nationally and 41.8% of households in Arbaminch town use inadequately iodized salt [1].

To a say a HH used a adequately iodized salt (the salt iodine content should > 15 ppm and < 40 ppm) and more than 90% of the population need to use this amount of iodized salt to declare as the iodized salt is available at the community [8]. Visible geographic variations of iodized salt availability in Bangladesh [5], Saudi Arabia [6], and other countries are observed. Other studies also pointed that the availability of iodized salt varies geographically across different countries [7]. Young and educated household head, poor and rural households significantly increase and decrease the household iodized salt availability in Bangladesh [5]. Different socioeconomic and household-level factors are associated with unavailability of iodized salt in different countries [1, 4, 5, 7, 9–24]. Age and level of education of household head, wealth index, place of residence, knowledge on iodized salt, marital status, family size, using packed salt [1, 4, 10, 11, 14, 25–27]. Evidenced from systematic review and meta-analysis, significant geographic variations of iodized salt availability were observed in Ethiopia with the highest in Addis Ababa to the lowest in Dire Dawa [1]. But these studies were not addressing the factors associated with the geographic variations.

Even though the iodized salt availability is low in Ethiopia, the spatial distribution and determinant factors were not identified. Hence, this study was aimed to assess the geographic variations and associated factors of un-iodized salt availability in Ethiopia using the Ethiopian Demographic and Health Survey (EDHS) 2016 data, which may help to achieve the universal salt iodization program by giving more emphasis on the identified hotspots of un-iodized salt availability in Ethiopia.

## Methods

*Study design and period* we conducted an in-depth secondary data analysis of the Ethiopian demographic survey 2016. The EDHS 2016 was a nationwide cross-sectional study conducted from January to June, 2016 [28].

*Study area* Ethiopia is a second most populous country in Africa next to Nigeria with more than hundred million peoples living in nine regions and two administrative cities. Nutritional deficiencies like iodine are common public health problems and more than one-tenth of the households use un-iodized salt [28].

**Fig. 1:**
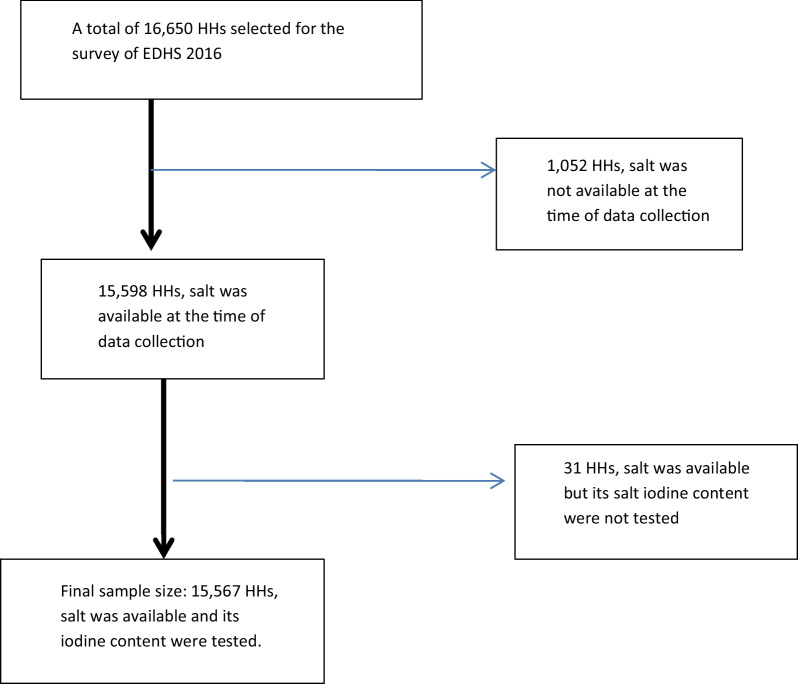
Data extraction process flow diagram. The horizontal arrows indicate the excluded households from the analysis. EDHS: Ethiopian Demographic and Health Survey, HHs: Households

*Data source and measurement* we used the EDHS 2016 household data containing 16,650 households that has been accessed after being registered as an authorized user from the major demographic and health survey (http://www.dhsprogram.com). The households were selected using a stratified multistage sampling technic. The major Demographic and Health Survey (DHS) collects survey on health and health related indicators across different countries. The EDHS 2016 is the fourth major survey in Ethiopia that collected health and health related indicators including household-level un-iodized salt availability. The household questionnaire was used to collect the un-iodized salt availability at the household level. The detail of the data collection and sampling procedure are available elsewhere [28]. A total of 16,650 households from 645 enumeration areas were included for the household characteristics. We included 15,567 households’ data for the final analysis to identify the determinant factors of iodized salt availability (See Fig. 1).

For the spatial analysis, study participants from the clusters where the GPS data were zero (21 clusters) were excluded. Finally, we used 420 and 202 clusters from the rural and urban areas, respectively.

*Study variables* The dependent variable was un-iodized salt. Independent variables were socio-demographic variables such as age of the household head, marital status, education, occupation, age, religion, region of residence, and wealth index. Here are definitions of some composite variables.

**Table:** 

Variable	Measurement
Hotspots of un-iodized salt (hot spot/cold spot)	Was considered if the clusters had Z-score greater than 1.96 using the Getis-Ord Gi* statistics
Un-iodized salt (Yes/no)	Was considered if the households salt Iodine shows negative
Health facility access (good/poor)	Was computed based on the distance from the women’s data. Above the median of the community of distance a big problem to health facilities is considered as communities’ poor health facility access
Altitude of residence (lowland/highland)	was defined below 2200 m above sea level as low land; 2200 m and above was considered as highland areas based on the median altitude of the community
Community media exposure (poor/good)	Was computed from the internet or listening television or reading newspaper at least once per week. It was computed to the community level and a community had low media exposure if they got below the median. Low if below the median of the community uses media at least once per week. High if median and above of the cluster uses media at least once per week

Other variables were directly used from the available data source and you can find them some elsewhere from the EDHS 2016 survey report [28].

*Data analysis* we accessed the data from the major DHS program; data cleaning, recoding, and weighting were conducted using Stata 16. The data were weighted using the household weighting variable (hv005) as per the recommendation of the major DHS program. We used Survey (svy) command for descriptive and analytical analysis. The detail of how to weigh the data found from the EDHS 2016 report Annex A [28]. The data were checked for missing values and codes before recoding the independent variables to maintain the integrity. For the spatial analysis, we computed the prevalence of un-iodized salt over the clusters and we used as an outcome variable. We tested the spatial autocorrelations using the global Moran’s index (*I*) by using an inverse distance with row standardization to balance the sampling errors among the enumeration areas (Clusters). Global Moran’s I is an autocorrelation coefficient with a value ranging from (− 1, 1) indicates the overall spatial clustering at the country level/general but cannot show where are these significant clusters found. If the value is close to − 1 this disease of interest/un-iodized salt utilization was spatially clustered while the reverse indicates spatial dispersion [2–4]. If the global Moran’s index was significant, further spatial analysis is required to identify local level significant clusters. The global Moran’s index was significant and we proceed the local level hotspot analysis using the Getis-Ord Gi* statistics. If the Getis-Ord Gi* statistics Z-score < − 1.96, between − 1.96–1.96 and above 1.96 implies significant cold spot (low risk), none significant and hotspot (high) risk clusters, respectively 5(5). As the data were based on the random samples taken, we estimate (predict) the magnitude of un-iodized salt using empirical Bayesian interpolation techniques.

For binary logistic regression to assess factors associated with un-iodized salt availability, Chi-square assumption was tested for each variable. Multi-collinearity was checked using standard errors and variance inflation factors. If standard error is > 2sd, the variance inflation factor greater than 10 multi-collinearity could be declared but no variable has this effect. The multilevel clustering effect was tested using the intra-class correlation coefficient and it was nearly zero (ICC = 0.00001). Hence, the hierarchical nature had no significant effect and we considered the binary logistics regression. The variables with *p*-value < 0.2 in the univariate analysis were considered for multivariate analysis. A *p*-value < 0.05 was used as a cutoff point to declare statistical significance and finally adjusted odds ratios (AOR) with 95% confidence interval (95% CI) was reported. The logistics regression model goodness of fit was tested using Hosmer and Lemeshow goodness-of-fit test and it produces a *P*-value of 0.96, and the model is adequately fit for the data.

## Results

The magnitude of un-iodized salt availability was 14.19% (95% CI; 13.65, 14.75) among Ethiopian households. We found that Afar region was a region with lowest iodized salt users while Oromia region accounts more than a one-third of the iodized salt user of the population (see Table 1).

**Table 1:** Regional variations of un-iodized salt in Ethiopia

Regions	Un-Iodized salt (%)	Total (%)
Tigray	139(12.30)	1130(7.01)
Afar	34(25.76)	132(0.83)
Amhara	342(8.41)	4069(25.53)
Oromia	470(8.06)	5829(36.57)
Somali	174(37.50)	464(2.92)
Benishangul	10(5.81)	172(1.08)
SNNPR	448(13.73)	3263(20.47)
Gambela	6(13.95)	43(0.27)
Harari	5(11.63)	43(0.27)
Addis Ababa	66(9.32)	708(4.44)
Dire Dawa	14(16.87)	83(0.52)

About 12,767 (81.1%) of the study participants were rural resident, 8703(54.6%) of the household heads had no education and 11,847 (74.33%) of the households found in highland (above 2200 m of sea level) areas of the country (see Table 2).

**Table 2:** Socioeconomic and Demographic characteristics of the study participants

Variable	Category	Total (%)
Age of the household head	13–30.99	4044(24.37)
	31–40.99	3651(22.91)
	41–56	4121(25.86)
	Above 56	4122(25.86)
Place of residence	Urban	3172(19.9)
	Rural	12,767(81.1)
Sex of the household head	Male	11,867(76.45)
	Female	4073(25.55)
Wealth index of the household	Poorest	3015(18.91)
	Poorer	3075(19.29)
	Middle	3041(19.08)
	Richer	2998(18.85)
	Richest	3811(23.91)
Current marital status	Never	646(4.05)
	Married	11,197(74)
	Widowed	1851(11.62)
	Divorced	1106(6.94)
Education level	No education	8703(54.6)
	Primary	4650(30.43)
	Secondary	1250(7.84)
	Higher	1089(6.83)
	Do not know	48(0.3)
Health facility access of the community	Poor	8341(52.33)
	Good	7598(47.67)
Community media exposure	Poor(low)	7450(46.74)
	Good	8489(53.26)
Altitude	Highland	11,847(74.33)
	Lowland	4091(25.67)

Age was classified based on the interquartile ranges

### Spatial distribution of un-iodized salt among households

**Fig. 2:**
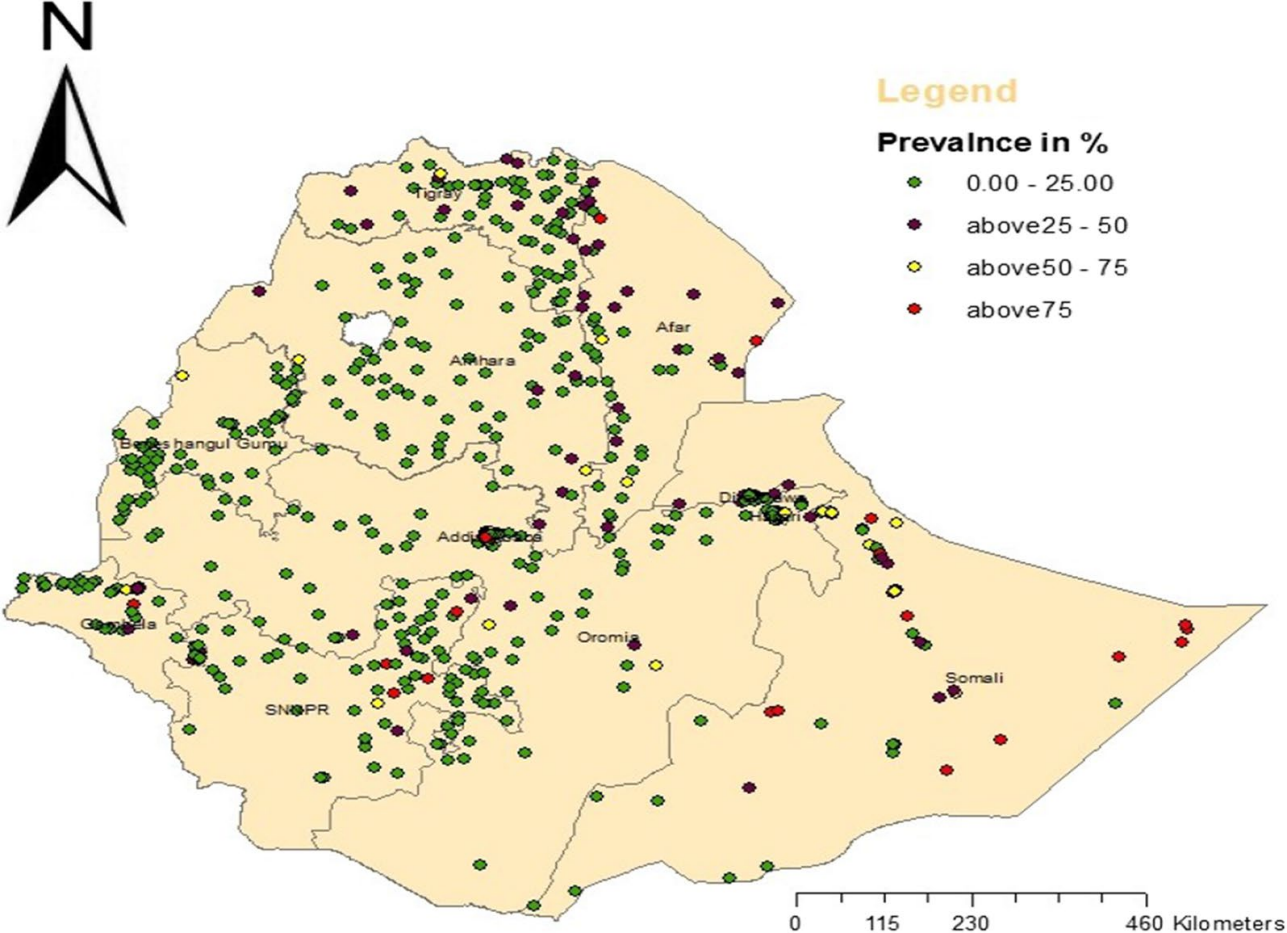
Spatial distribution of un-iodized salt in Ethiopia

The distribution of un-iodized salt varies across regions. Enumeration areas with low availability (< 25% of HH with un-iodized salt) of un-iodized salt were found in Amhara, Tigray, most parts of Oromia, Addis Ababa, and Benishangul Gumuz, while clusters with high prevalence (> 75% of HH with un-iodized salt) of un-iodized salt were found in Afar and Somali regions (see Fig. 2).

**Fig. 3:**
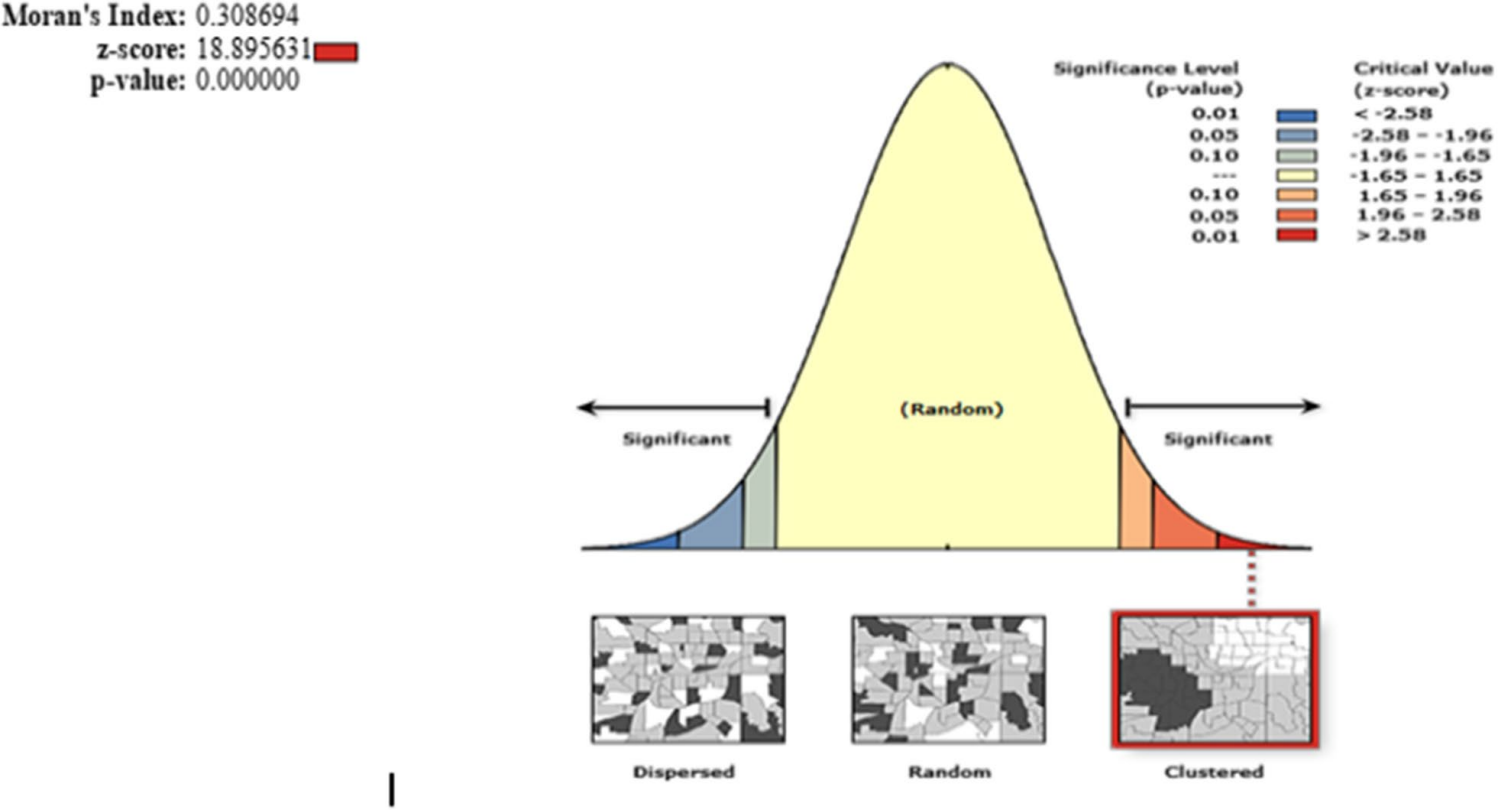
Spatial autocorrelation of un-iodized salt among households of Ethiopia using a global Moran’s index

We found a significant geographic variation of un-iodized salt availability among households of Ethiopia at national level with a global Moran’s’ Index (*I* = 0.31 with *P*-value < 0.001 and Z-score of 18.90), which shows that the distribution of un-iodized salt was spatially clustered at the national level and further local level test of spatial clustering was required (see Fig. 3).

**Fig. 4:**
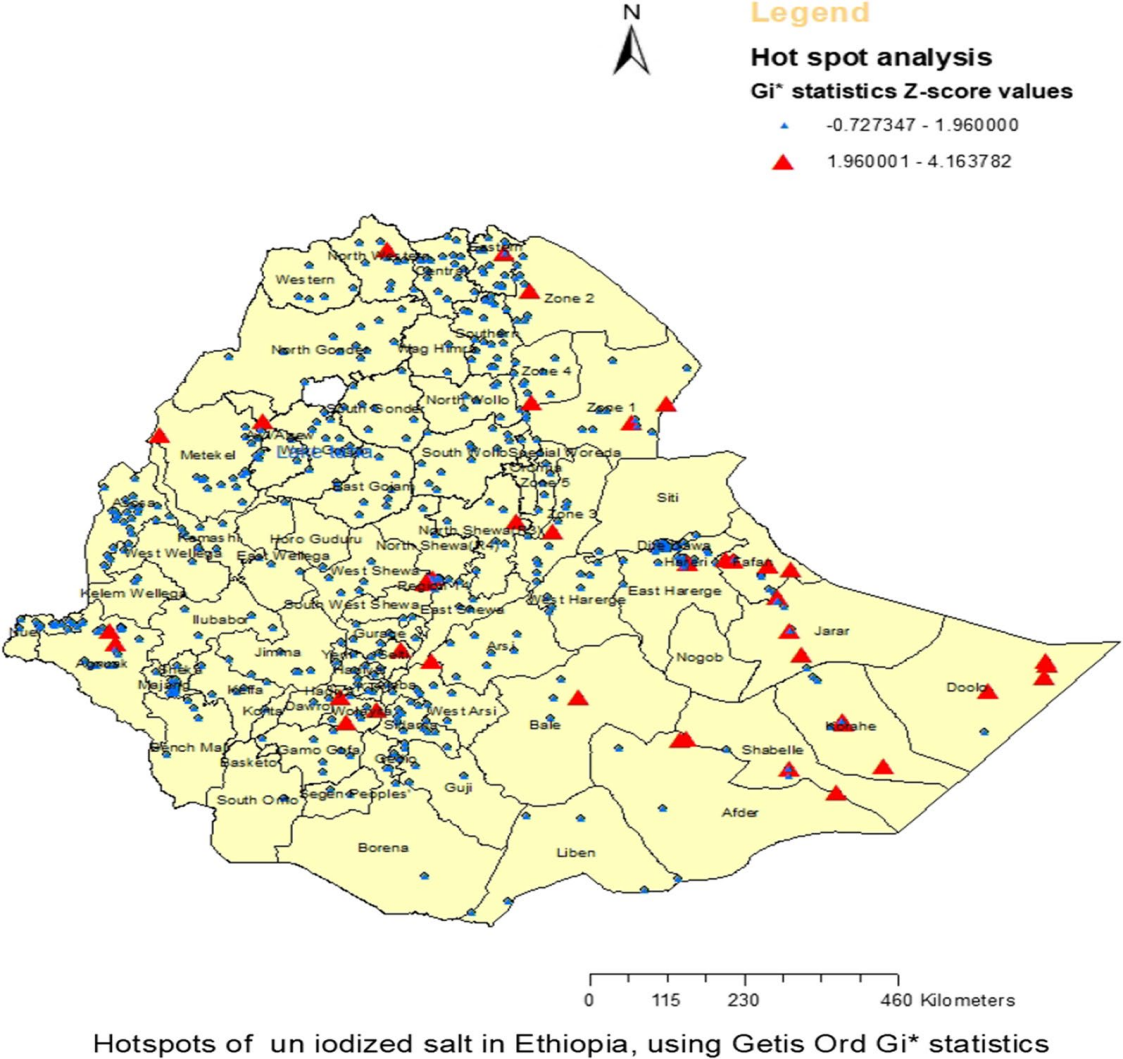
Hotspot analysis of household-level un-iodized salt availability in Ethiopia using Getis-Ord Gi* statistics. A dot represents one cluster (Enumeration area) and we labeled the map using the zonal level of the shape file

The hotspot analysis pointed that majority of the clusters were not significant. Hotspot clusters were found in zones of Somali, Afar, and South Nations Nationalities and people’s regions (SNNPR) of the country (see Fig. 4).

**Fig. 5:**
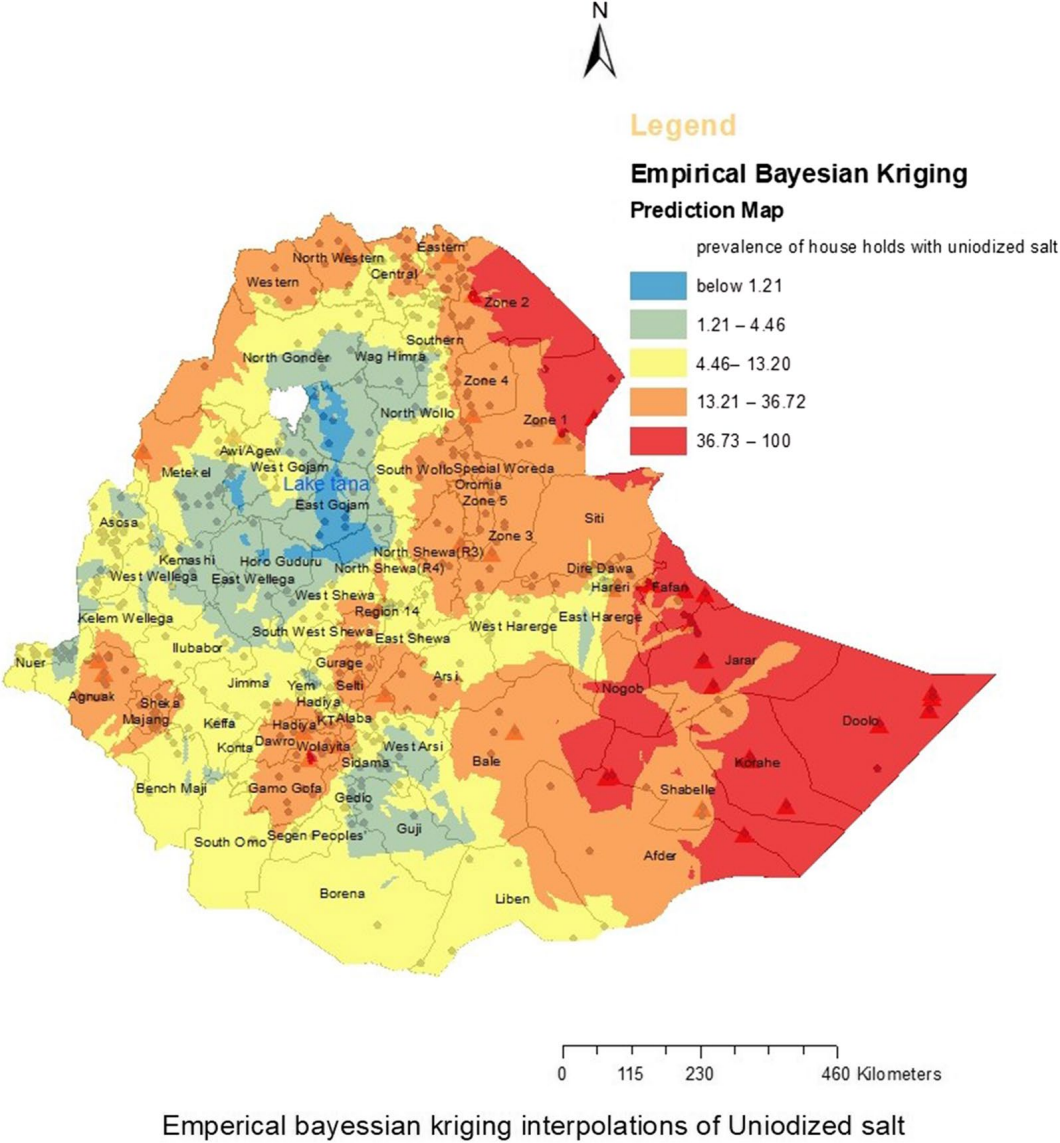
Empirical Bayesian interpolation of household-level un-iodized salt availability in Ethiopia predication map

We predict the magnitude of the un-iodized salt using empirical Bayesian interpolation techniques. Similar to the hotspot analysis, the prediction map shows most of the Afar and Somali regions with high prevalence of un-iodized salt which showed consistency of the predicted and existing magnitudes of un-iodized salt across different districts (See Fig. 5).

### Associated factors of un-iodized salt among households

Among used variables, the wealth index, educational level of the household head, and altitude of residence were significantly associated with households’ un-iodized salt availability. Compared to households with poorest wealth index; the households having poorer [45% (AOR = 0.55, 95% CI: 0.48, 0.64)], middle [49% (AOR = 0.51, 95% CI: 0.44, 0.60)], richer [45% (AOR = 0.55, 95% CI: 0.47, 0.64)], and richest [39% (AOR = 0.61, 95% CI: 0.50, 0.75) times less odds of having un-iodized salt.

Considering the household heads level of Education, those who had a secondary and above secondary education had 28% (AOR = 0.72, 95% CI: 0.60, 0.67) and 46% (AOR = 0.54, 95% CI: 0.43, 0.67) less odds of having un-iodized salt as compared to uneducated household heads, respectively. Those households living in a community of above 2200 m altitude above sea level had 16% (AOR = 1.16, 95% CI: 1.05, 1.29) higher odds of having un-iodized salt compared to their counterpart (See Table 3).

**Table 3:** Binary logistics regression analysis of un-iodized salt availability among Ethiopian households using the Ethiopian Demographic and Health Survey 2016 data

Variables	Category of the variables	COR (95% CI)	AOR (95% CI)
Place of residence	Urban	1	1
	Rural	1.57(1.41,1.74)	1.18(0.96,1.44)
Sex of the household head	Male	1	1
	Female	1.11(1.01, 1.22)	1.05 (0.93,1.19)
Wealth index of the household	Poorest	1	1
	Poorer	0.54(0.47, 0.62)	0.55(0.48, 0.64)*
	Middle	0.50(0.42, 0.58)	0.51(0.44, 0.60)*
	Richer	0.51(0.44, 0.60)	0.55(0.47, 0.64)*
	Richest	0.45(0.40, 0.50)	0.61(0.50, 0.75)*
Marital status	Never	1	1
	Married	1.26(1.01,1.58)	0.86(0.68, 1.10)
	Widowed	1.42(1.10, 1.82)	0.87(0.67, 1.15)
	Divorced	1.21(0.92, 1.59)	0.82(0.62, 1.10)
Education level	No education	1	1
	Primary	0.77(0.70, 0.86)	0.90(0.80, 1.002)
	Secondary	0.59(0.49, 0.70)	0.72(0.60, 0.87)*
	Higher	0.43(0.35, 0.52)	0.54(0.43, 0.67)*
	Do not know	0.59(0.25, 1.39)	0.67(0.29, 1.58)
Communities’ health facility access	Distance big problem	1	1
	Distance not a big problem	0.89(0.81, 0.98)	0.93(0.84, 1.03)
Communities’ media use	Low	1	1
	High	1.06(0.98, 1.17)	1.00(0.91, 1.11)
Communities’ altitude of residence	Below 2200 m above sea level	1	1
	2200 and above	1.18(1.07, 1.30)	1.16(1.05, 1.29)*

Goodness-of-fit test using Hosmer–Lemeshow test of goodness (*p*-value = 0.96)

*AOR* Adjusted odds ratio, *COR* Crude Odds Ration

*Statistically significant factors

## Discussions

This study was undertaken to assess the spatial distribution and determinants of un-iodized salt availability among households of Ethiopian households. The overall availability of iodized salt in Ethiopia was low as compared to other countries elsewhere [2, 9, 12, 16, 19, 29–35] and the utilization of un-iodized salt remains high as public health problem. This may cause a number of iodine deficiency-related complications including Goiter and other cardiovascular problems.

Significant hotspots of un-iodized salt were found in most parts of Somali, Afar, and Benishangul Gumuz regions of Ethiopia. This finding was comparable with other systematic review and meta-analysis reports where the availability of un-iodized salt varies significantly across regions of Ethiopia [36]. Another study pointed that the existence of significant regional variations in access to iodized salt across 11 lower income countries [29]. The possible justifications for the spatial variations of un-iodized salt in Ethiopia might be different across regions; Afar and Somali regions were the places where most hotspots of un-iodized salt users found and this might be explained by the reasons given herewith. For Afar and Somali regions, there are places used for local and un-iodized salt extraction including the Afdera and Asale lacks and other lands in Somali [37, 38]. These local dwellers extract/produce un-iodized salts to other parts of the country. Evidences show that the transportation and iodization cost was high as compared to local produced salts and small (local) producers can compete with low price and none-iodized salt [3, 39]. These local small-scale producers have a challenged the salt trade in Sub-Saharan Africa and this might works true for afar region where a number of small producers extract none-iodized salt and sell with cheap price for local residents of the region [3, 40]. Even lack of commitment by the local salt producers to iodize, the harsh weather in Afdera, low productivity of the iodization machines challenges Ethiopia to have adequately iodized salt [41].

Having better wealth index reduces the households’ odds of un-iodized salt utilization as compared to these households with poorest wealth index. This evidence is supported by other studies conducted in Ethiopia [1, 14, 42, 43], Bangladesh [5], and Tanzania [13] where improved wealth index reduces the odds of households utilization of iodized salt. This might be due to accessibility and cost of the iodized salt is more acceptable by the wealthier than the poorest [3, 4, 7, 41]. As it is known, iodized salt is more expensive than un-iodized one due to the cost of transportation and iodization process and the poor may access locally produced un-iodized salts in low price [3, 41].

Compared to uneducated household heads, those with secondary and above secondary educations had 28 and 46% less odds of using un-iodized salt, respectively. These findings are supported by reports of studies conducted in Ethiopia different parts of Ethiopia [14, 25, 44], Sub-Saharan Africa [4], and Bangladesh [5]. This might be explained by those who are educated may have better knowledge about the benefits of salt iodization and risks of iodine deficiency disorders [45].

Considering the altitude of residence, we found that living in high land areas above 2200 m increases the odds of un-iodized salt availability and we could not find any study considering altitude as significant predictors of un-iodized salt. And this might be due to the fact that most of the highland areas are difficult to reach areas for different goods and services. Iodized salt transportation is one of the challenges of its utilization by different communities [3, 29].

*Limitations* we used a secondary data and we are not immune of the limitations of secondary data. The geographic distortions of the coordinate data for the security purpose may limit us to determine the specific locations accurately. Unavailability of behavioral factors, women cooking practices, and their knowledge and practice about iodized salt were missed to clearly interpret the utilization.

*Conclusions and recommendations* Household-level un-iodized salt availability was spatially clustered. The majority of significant hotspot clusters were found in Somali, Benishangul Gumuz, and Afar regions. Better wealth index and education of the household heads reduces the odds of un-iodized salt availability while living in a high altitude above 2200 m increases the odds of un-iodized salt availability in Ethiopia. Giving more emphasis on hotspot clusters and improving the household heads' education and wealth index might reduce the availability of un-iodized salt in Ethiopia.

## Data Availability

The data used for preparation of this manuscript are available from http://www.dhsprogram.comand anyone can access through online request as authorized user. The authors prepared the data that were used for preparation of this manuscript can be shared if required. The shape files of the maps were freely available without any restriction at Open Africa (https://africaopendata.org/dataset/ethiopia-sha.pefiles).

## Abbreviations

AOR: Adjusted odds ratio
CI: Confidence interval
EDHS: Ethiopian Demographic and Health Survey
EA: Enumeration area
HH: Households
